# Health‐Related Behaviour Clusters and Functional Dentition in Older People

**DOI:** 10.1111/ger.12807

**Published:** 2025-01-08

**Authors:** Fatimah Alobaidi, Ellie Heidari, Wael Sabbah

**Affiliations:** ^1^ Faculty of Dentistry, Oral & Craniofacial Sciences King's College London London UK

**Keywords:** adult, dentition, health behaviour, social factors, tooth loss

## Abstract

**Objectives:**

To identify different clusters of health‐related behaviours and examine whether these clusters are associated with maintaining 20 or more teeth.

**Background:**

Engaging in risky behaviours impacts tooth loss, particularly among older adults. Maintaining 20 teeth is a challenge for this age group. The co‐occurrence of health‐risk behaviours is common and has been linked to an increased risk of multiple chronic diseases, including tooth loss.

**Material and Methods:**

A cross‐sectional analysis of wave 7 of the English Longitudinal Study of Ageing (ELSA) was conducted. Functional dentition was self‐reported as having 20 or more teeth. Four health‐related behaviours (smoking, alcohol intake, fruit and vegetable consumption, and physical activity) were analysed to investigate their association with functional dentition. Demographic characteristics (sex, age, ethnicity) and socioeconomic factors (education, wealth) were included as covariates. Latent Class Analysis (LCA) was conducted using four dichotomised behaviour variables to identify clusters of behaviours. Logistic regression modelling was used to examine the association between clusters of health‐related behaviours and functional dentition. The model was adjusted for demographic and socioeconomic factors.

**Results:**

A total of 7783 participants were included. The LCA model identified three clusters: (1) risky, (2) moderate and (3) healthy. In the fully adjusted logistic regression model, the odds of having a functional dentition were 1.42 higher among those in the moderate cluster (95% CI: 1.23, 1.65), and 1.70 higher among those in the healthy cluster (95% CI: 1.39, 2.09) than for participants in the risky cluster.

**Conclusion:**

Risky behaviours tend to cluster among older adults. Engaging in multiple risky behaviours is associated with having fewer than 20 teeth. Initiatives and public health campaigns that focus on these clustering patterns, as well as the underlying factors, could benefit both oral and general health.

## 1 Introduction

The World Health Organisation (WHO) defines a functional dentition as having 20 or more natural teeth that are aesthetically pleasing and functionally intact for the whole of a person's life without requiring prosthodontic replacements [1]. Having a functional dentition is crucial for several reasons. It can prevent any damage to the temporomandibular joint, reduce functional limitations, and enhance the stability of the occlusion [2]. Additionally, it has a significant impact on an individual's quality of life, influencing their physical health and social well‐being [3, 4]. Tooth loss is the result of a complex interaction between several biological, psychological, and social factors [5, 6]. The main biological causes of tooth loss are periodontal disease and dental caries [7]. Several factors can lead to tooth loss by increasing the risk of dental diseases [8, 9] including lower socioeconomic status [10], inaccessibility of dental care [11], negative attitudes toward oral health [12] and engaging in risky behaviours [13].

Risky behaviours can be defined as any acts that harm or delay healing [14]. Various behaviours including alcohol drinking, smoking, lower consumption of fruit and vegetable, and poor physical activity have a serious effect on overall health, with chronic diseases being one of the many [15, 16, 17]. In addition to general health, these behaviours can negatively impact on oral health [18, 19]. Smoking and alcohol intake significantly impact tooth loss and functional dentition by promoting periodontal inflammation, bone loss, tooth decay and oral infections [20, 21, 22]. Other behaviours that are not directly linked to oral health may also impact functional dentition. Physical activity and consumption of fruits and vegetables can contribute to optimal oral health by supporting gingival tissue [23], reducing inflammation [24], and providing essential nutrients [25, 26]. These healthy lifestyle choices play vital roles in maintaining healthy oral tissues and teeth. However, risky behaviours often occur in groups and clusters [27, 28], with individuals who have poorer oral health being more likely to engage in various risky behaviours [29, 30].

Different studies have explored clusters of health‐related behaviours among children and adolescents in relation to dental caries [29, 30]. The negative impact of these behaviours implies that they may also be linked to tooth loss and affect functional dentition. A previous study using longitudinal data found that a combination of oral hygiene behaviours (tooth brushing, flossing, and professional prophylaxis) was associated with maintaining teeth among older adults [31]. However, the study did not include behaviours not directly related to oral health, such as physical activity, smoking or alcohol consumption. To the best of our knowledge, no study has examined the effect of clusters of health‐related behaviours on functional dentition among older adults.

Identifying specific clusters of health‐related behaviours related to functional dentition could provide a more comprehensive understanding of the factors that affect tooth loss. Accordingly, the aim of this study is to identify different clusters of health‐related behaviours among older English adults and to determine if the clustering of behaviours influences the maintenance of 20 teeth or more among older English adults.

## 2 Materials and Methods

### 2.1 Study Sample

This is a cross‐sectional analysis of wave 7 (2014/2015) of the English Longitudinal Study of Ageing (ELSA) [32]. The ELSA is an English prospective observational study of adults aged 50 years and older. Participants from the Health Survey for England in 1998, 1999, and 2001 were the source for ELSA. In the survey, information on mental, physical and social health was collected using a computer‐assisted personal interview (CAPI) and a self‐completion questionnaire (SCQ). Oral health was measured every 2 years in wave 3 (2006/07), wave 5 (2010/11), and wave 7 (2014/15). Wave 7 was the only wave that measured the number of remaining teeth, with 9666 participants completing the interviews in person. All participants have provided written informed consent, and all ELSA waves received ethical approval from the Multicentre Research and Ethics Committee (MREC/01/2/91).

### 2.2 Assessment of Outcome

Functional dentition was assessed during the CAPI with the question “how many natural teeth have you got?” with responses: “none at all, between 1 and 9 natural teeth, between 10 and 19 natural teeth, 20 or more natural teeth”. This variable was dichotomised into having a functional dentition (20 teeth or more), and not having a functional dentition (fewer than 20 teeth).

### 2.3 Assessment of Exposure

Four behaviours (smoking, alcohol intake, fruit and vegetable consumption, and physical activity) were collected during the CAPI and SCQ. All behaviours were used as dichotomous variables. Smoking status was determined from the questions “do you ever smoke” and “are you a current smoker”. The variable was categorised as never (never/former smokers) versus smoker (current smoker). Alcohol intake was collected from questions regarding the number of drinking units of different types of alcohol (spirits, wine, beer, lager or cider). The variable was then categorised as more than 14 units (high consumption) versus 14 units or less per week (no to moderate consumption) [33]. Fruit and vegetable consumption was assessed through questions about the portions of fruit and vegetables consumed daily. The variable then was categorised as less than 5 portions versus 5 or more portions per day. Physical activity was determined from the question “does the participants take a part in a vigorous, moderate, or mild activity” with response: (more than once a week, once a week, one to three times a month, hardly ever or never). The variable was then categorised as no or mild activity versus moderate or high activity.

### 2.4 Covariates

Demographic data including sex, age and ethnicity, and socioeconomic factors (education and wealth) were collected during the CAPI. Age was used as a continuous variable. Ethnicity was categorised as white or non‐white. Education was categorised as no education or lower (less than O‐levels), medium level of education (O‐levels), or higher education or A levels (higher). Wealth quantiles were created using total non‐pension wealth, with 1 being the poorest and 5 the wealthiest. The total wealth calculation accounted for the total value of housing, financial assets and physical possessions owned by the household, excluding any debts [34].

### 2.5 Statistical Analysis

All analyses were conducted using Stata 17. Latent Class Analysis LCA was conducted using four dichotomous behaviours. In LCA, a class‐specific response probability is computed that shows the likelihood of a participant from a certain cluster exhibiting a specific behaviour [35]. Goodness‐of‐fit indices such as the Akaike Information Criterion (AIC) and the Bayesian Information Criterion (BIC), which shows the adjusted value for the log‐likelihood, were used to determine the optimal number of clusters. Logistic regression modelling examined the association between clusters of health‐related behaviours and functional dentition. The model included demographic characteristics (sex, age, ethnicity) and socioeconomic factors (education, wealth). Stata survey commands were used throughout the analysis accounting for sample weights.

## 3 Results

After the exclusion of participants with missing information (*n* = 1883), a total of 7783 complete participants were included in this analysis.

**FIGURE 1:**
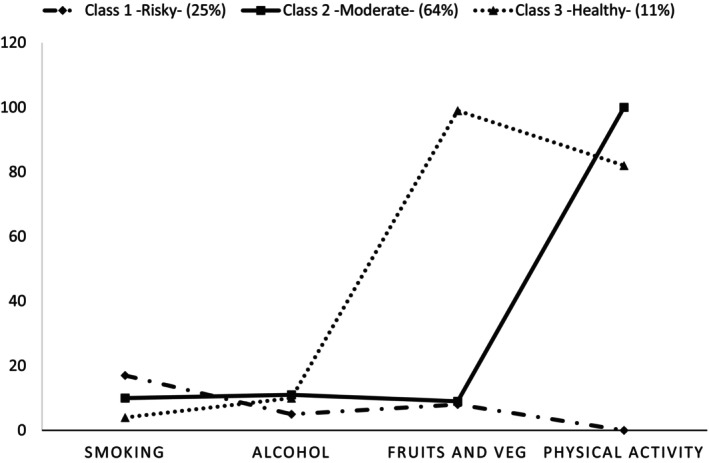
Cluster‐specific probabilities of health‐related behaviours for the three‐cluster model (*n* = 7783).

One to four class models were generated using LCA with four health‐related behaviours. The three‐class model was the best match for the data, according to the goodness‐of‐fit indices (AIC and BIC). As shown in Figure 1, the weighted percentages were 25% for class 1, 64% for class 2 and 11% for class 3.

The estimated probabilities of health‐related behaviours specific to each cluster in the three‐cluster model from the LCA (Figure 1) indicate that class 1 had the highest probability of smoking and the lowest physical activity, and fruit and vegetable consumption (risky cluster). Class 2 had the highest probability of physical activity and moderate probabilities in all other behaviours (moderate cluster). Class 3 had the highest probability of fruit and vegetable consumption and the lowest probability of smoking (healthy cluster).

The characteristics of the included, excluded, and baseline ELSA sample are displayed in Table 1. In the included sample, 65.9% of the participants had a functional dentition, and 52% were female, with a mean age of 66.4. Almost half the participants (43%) had obtained less than an O‐level education. Most of the excluded sample had missing information on wealth, and some missing on education. In the excluded sample, 77.4% of participants had functional dentition and 53% were female, with a mean age of 62.

**TABLE 1:** Characteristics of all variables used in the analysis of ELSA wave 7 (2014/15) (study sample and excluded sample), and baseline sample (wave 1).

	Study sample		Excluded sample		Baseline sample of ELSA	
	Wave 7 (2014/15)		Wave 7 (2014/15)		Wave 1 (2002/03)	
	(*n* = 7783)		(*n* = 1883)		(*n* = 12,099)	
Variables	%, Mean	95% CI	%, Mean	95% CI	%, Mean	95% CI
Functional dentition						
Yes	65.9%	(64.7, 67.1)	77.4%	(76.1, 79.8)		
No	34.1%	(32.9, 35.2)	21.8%	(20.2, 23.9)		
Missing			0.8%			
Clusters						
Risky	25.4%	(24.2, 26.5)				
Moderate	63.1%	(61.8, 64.4)				
Healthy	11.5%	(10.7, 12.4)				
Age	66.4	(66.1, 66.7)	62.0	(61.52, 62.47)	64.2	(63.9, 64.4)
*p*		< 0.001				
Gender						
Male	47.1%	(45.7, 48.4)	46.3%	(44.0, 48.5)	45.7%	(44.7, 46.6)
Female	52.9%	(51.6, 54.3)	53.7%	(51.5, 56.0)	54.3%	(53.4, 55.3)
Ethnicity						
White	94.7%	(93.9, 95.5)	93.0%	(92.3, 94.6)	96.4%	(96.4, 97.0)
Non‐white	5.3%	(4.5, 6.1)	6.5%	(5.4, 7.7)	3.0%	(2.5, 3.0)
Missing			0.4%		0.6%	
Education						
Less than o‐level	43.1%	(41.8, 44.4)	30.0%	(33.6, 38.6)	54.6%	(55.4, 57.4)
o‐level	29.2%	(27.9, 30.4)	26.3%	(29.5, 34.1)	22.6%	(20.8, 22.3)
Higher	27.7%	(26.5, 28.9)	26.6%	(29.9, 43.4)	22.5%	(21.1, 22.7)
Missing			17.1%		0.3%	
Wealth						
1	19.9%	(18.8, 21.1)	2.3%	(15.9, 28.2)	18.0%	(18.7, 20.1)
2	20.1%	(19.0, 21.2)	2.1%	(14.7, 25.8)	18.5%	(19.2, 20.7)
3	19.9%	(19.0, 21.0)	2.4%	(17.3, 29.4)	18.5%	(19.2, 20.7)
4	19.6%	(18.8, 20.9)	2.0%	(13.4, 24.6)	18.5%	(19.3, 20.8)
5	21.0%	(19.2, 21.2)	1.6%	(11.2, 21.4)	19.5%	(19.8, 21.3)
Missing			89.6%		7.5%	

Table 2 present the characteristics of the included variables by a cluster of health‐related behaviours and functional dentition. The mean age of participants with and without functional dentition was 63 and 72, respectively. Functional dentition was lowest among participants in the risky cluster (40.5%), with the lowest education (34.8%) and lowest wealth (46.1%). Most of the participants in the moderate and healthy clusters had 20 teeth or more and had higher education and greater wealth. Most of the participants in the risky cluster had less than 20 teeth with less than an O‐level of education and had the lowest wealth.

**TABLE 2:** Cluster of health‐related behaviours and functional dentition by sociodemographic characteristics, ELSA wave 7 (2014/25) (*n* = 7783).

	Clusters						Functional dentition			
	Risky		Moderate		Healthy		Yes		No	
	Percentage/Mean	95% CI	Percentage/Mean	95% CI	Percentage/Mean	95% CI	Percentage/Mean	95% CI	Percentage/Mean	95% CI
Functional dentition										
Yes	17.5%	(16.2, 18.8)	69.3%	(67.7, 70.8)	13.2%	(12.1, 14.5)				
No	40.6%	(38.6, 42.5)	51.3%	(49.3, 53.3)	8.2%	(7.2, 9.2)				
Clusters										
Risky							45.5%	(42.9, 48.0)	54.5%	(51.5, 57.1)
Moderate							72.3%	(70.9,73.7)	27.67%	(26.3, 29.1)
Healthy							75.8%	(72.8, 78.6)	24.1%	(21.4, 27.2)
Age	71.8	(71.1, 72.5)	64.4	(64.1, 64.8)	65.5	(65.0,66.3)	63.2	(62.9, 63.6)	72.5	(72.0, 73.1)
Gender										
Male	21.0%	(19.4, 22.6)	67.7%	(65.8, 69.6)	11.3%	(10.1, 12.7)	66.4%	(64.9, 68.1)	33.6%	(31.8, 35.5)
Female	29.3%	(27.7, 30.9)	59.0%	(57.3, 60.7)	11.7%	(10.6, 12.8)	65.5%	(63.5, 67.1)	34.5%	(32.9, 36.1)
Ethnicity										
White	24.9%	(23.8, 26.0)	63.4%	(62.1, 64.7)	11.7%	(10.9, 12.6)	65.1%	(63.9, 66.3)	34.9%	(33.7, 36.1)
Non‐white	34.5%	(27.8, 41.9)	57.5%	(49.8, 64.9)	7.9%	(4.8, 12.9)	80.6%	(75.7, 67.1)	19.3%	(14.79, 24.9)
Education										
Less than o‐level	34.9%	(33.0, 36.8)	56.1%	(54.1, 58.0)	9.0%	(8.0, 10.2)	53.3%	(51.3, 55.2)	46.7%	(44.8, 48.7)
o‐level	21.6%	(19.6, 23.7)	66.1%	(63.7, 68.5)	12.3%	(10.6, 14.2)	71.3%	(69.1, 73.3)	28.7%	(26.6, 30.9)
Higher	14.6	(13.0, 16.3)	70.9%	(68.6, 73.0)	14.5%	(12.8, 16.4)	79.9%	(78.2, 81.6)	20.1%	(18.4, 21.8)
Wealth										
Lowest quantile	46.1%	(42.9, 49.3)	49.1%	(45.9, 52.4)	4.7%	(3.7, 6.1)	47.8%	(44.5, 51.1)	52.2%	(48.9, 55.5)
Second lowest	29.4%	(26.8, 32.2)	61.3%	(58.3, 64.3)	9.2%	(7.5, 11.2)	60.7%	(57.7, 63.6)	39.3%	(36.4, 42.3)
Middle quantile	23.1%	(20.9, 25.4)	62.4%	(59.6, 65.1)	14.5%	(12.4, 16.9)	64.0%	(61.4, 66.6)	35.9%	(33.4, 38.6)
Second highest	16.9%	(15.0, 18.9)	70.1%	(67.5, 72.6)	13.0%	(11.2, 15.1)	74.9%	(72.7, 77.0)	25.1%	(23.0, 27.3)
Highest quantile	11.4%	(9.8, 13.3)	72.5%	(70.1, 74.9)	16.0%	(14.1, 18.1)	81.9%	(79.9, 83.8)	18.0%	(16.2, 20.0)

In the crude regression model (Table 3), the odds of having functional dentition were 3.1 higher in the moderate cluster (95% CI 2.8, 3.5) and 3.76 higher in the healthy cluster (95% CI 3.1, 4.5) more than the participants in the risky cluster (Table 3). After adjustment for covariates, the odds of having a functional dentition attenuated to 1.4 (95% CI 1.2, 1.6), and 1.70 (95% CI 1.4, 2.1) among those in the moderate and healthy clusters, respectively. A sensitivity analysis was conducted using a single behaviour instead of a cluster of behaviour, and similar results were observed (Data S1).

**TABLE 3:** Logistic regression model for having a functional dentition among older English adults, ELSA wave 7 (2014/15) (*n* = 7783).

Clusters	Crude model		Adjusted model	
	OR	95% CI	OR	95% CI
Risky	1.0	—	1.0	—
Moderate	3.1	(2.8, 3.5)***	1.4	(1.2, 1.6)***
Healthy	3.8	(3.1, 4.5)***	1.7	(1.4, 2.1)***
Age	0.9	(0.9, 0.9)***	0.9	(0.9, 0.9)***
Gender				
Male	1.0	—	1.0	—
Female	0.9	(0.9, 1.1)	1.2	(1.1, 1.4)***
Ethnicity				
White	1.0	—	1.0	—
Non‐white	2.2	(1.6, 3.1)***	1.9	(1.4, 2.7)***
Education				
Less than o‐level	1.0	—	1.0	—
o‐level	2.2	(1.9,2.5)***	1.3	(1.1, 1.5)***
Higher	3.5	(3.1, 4.0)***	1.0	(1.7, 2.3)***
Wealth				
Lowest quantile	1.0	—	1.0	—
Second lowest	1.7	(1.4, 2.0)***	1.7	(1.4, 2.2)***
Middle quantile	1.9	(1.6, 2.3)***	2.5	(2.1, 3.1)***
Second highest	3.3	(2.7, 3.9)***	3.9	(3.2, 4.7)***
Highest quantile	5.0	(4.1, 5.1)***	4.9	(4.0,6.1)***

*Note:* Adjusted model: adjusted for sociodemographic characteristic (sex, age, ethnicity, wealth, education).

*** *p* < 0.001.

## 4 Discussion

The study explored clusters of four health‐related behaviours, including smoking, alcohol intake, fruit and vegetable consumption, and physical activity among older English adults (aged 50 years and older). There was a significant difference in functional dentition between participants in the three clusters (risky, moderate, healthy) with those in the risky cluster being at higher risk of having fewer than 20 teeth. This relationship persisted even after accounting for socioeconomic factors.

Previous studies have discussed clusters of oral and general behaviours among different populations [28, 36, 37, 38, 39]. Most of these studies explored how health behaviours cluster together among the same populations and demonstrated that people with health‐compromising behaviours usually cluster together, with socioeconomic factors being the strongest predictor of these clusters.

Few studies have assessed clusters of behaviours in relation to oral health, particularly among older adults. Only one study has shown that the use of multiple oral hygiene practices was positively associated with tooth retention among older adults, in both cross‐sectional and longitudinal data [31]. These findings are similar to the current results, which clearly demonstrated that individuals with more favourable behaviours have a higher probability of having a functional dentition in old age. The present study uniquely examined four health‐related behaviours including fruit and vegetable consumption and physical activity, which are not directly linked to oral health.

The association between smoking and tooth loss has been established in previous research [20, 21]. It has been argued that smoking influences tooth retention through its impact on periodontal disease [40]. The use of nicotine or cigarette smoking may expedite the loss of alveolar bone [41] and smoking cessation could potentially contribute to the preservation of both periodontal and alveolar bone health [42]. In the current study, participants with the highest probability of smoking were more likely to engage in multiple risky behaviours and have fewer than 20 teeth.

The relationship between physical activity and tooth loss is not as clear. However previous studies have found that physical activity was associated with various behaviours, such as better oral hygiene practices [23], which are linked to positive general health practices and favourable oral health outcomes [43]. Exercising may promote behavioural change by motivating individuals to take care of their health and improve their oral hygiene [44]. In this study, being physically active was more common among those who exhibited healthy to moderate behaviours and also among those who had 20 teeth or more.

The fruit and vegetable consumption association with tooth loss is less obvious. Some argue that incorporating fruits and vegetables into daily diet could lead to a significant improvement in oral health status and reduce gingival inflammation [25, 26]. This study demonstrated that incorporating more than 5 portions of fruits and vegetables in your lifestyle was higher among those who have healthy behaviours and among those with 20 teeth or more.

The association between alcohol intake and tooth loss is complicated. While some researchers suggested that an increase in alcohol intake could lead to tooth loss and periodontal diseases [45], others have argued that alcohol intake could have a protective effect [22]. In the current study, alcohol intake was high among individuals in the moderate cluster with moderate probabilities of other risky behaviours; however, the chances of having 20 teeth or more were also high. One possible explanation for the protective effect of alcohol against tooth loss is the likelihood that individuals may choose alcoholic beverages over other options like sugar‐sweetened drinks [46]. Another contributing factor could be the type of alcoholic beverage consumed. Previous evidence has indicated a correlation between specific types of alcoholic drinks and social status, with wine consumption associated with higher socioeconomic status [47] and healthier eating habits [48]. Consequently, the observed protective effect of alcohol on tooth loss might be attributed to the higher socioeconomic levels and healthier lifestyle of individuals rather than being solely a consequence of alcohol consumption [49].

Although the study did not examine these behaviours, there are various behaviours that might be strongly associated with functional dentition and tooth loss, such as oral hygiene practices and dental visits. A previous study examined the use of multiple oral hygiene behaviours (toothbrushing, flossing and professional dental prophylaxis) and linked these behaviours to tooth retention [31]. The use of multiple oral hygiene practices can help maintain healthy gingival tissue [50], thereby enhancing the retention of natural teeth throughout adult life. Another behaviour that is linked to maintaining healthy teeth is regular dental visits [51]. Dental visits provide preventive care, which can play a major role in reducing the risk of complex problems and helping to maintain natural teeth. Tooth loss is the result of a cumulative effect of social factors on behaviours [52]. However, this was not tested in this study and therefore, we cannot conclude that tooth loss is solely attributable to behavioural factors.

There are a few limitations of the study worth mentioning. First, although the current study used data from a longitudinal survey, the number of teeth was only available in wave 7. This led to the use of cross‐sectional data, which does not support conclusions on causality or temporality. Second, while the survey includes various health‐related behaviours, it notably excludes significant behaviours like oral health practices and dental visits, which may have a stronger correlation with dental status, particularly the number of teeth. Third, self‐reported data were used in this study, which could be subjected to measurement bias (such as recall and social desirability bias). Nevertheless, in epidemiological studies, self‐reported measures are commonly employed due to their cost‐effectiveness and practicality, and they are widely accepted. Fourth, it was not possible to examine specific ethnic differences, as the survey removed this information to protect participant identity.

The strengths of the study include the use of latent class analysis, which provides accurate insights into the complex relationships between health‐related behaviours [53]. Moreover, the use of health‐related behaviours rather than oral behaviours is also an advantage, as it shows the influence of general behaviours on oral health outcomes. Additionally, the study used a community‐based sample that included a large population which limits the chances of systematic errors (selection bias).

The study has some implications. Risky behaviours impact both oral and general health, and often appearing together in the same individuals. Therefore, the findings support the underlying theory of the common risk factor approach and suggest addressing clustering of behaviours. This, in turn, prompts the integration of both oral and general health initiatives through a comprehensive health promotion strategy [54]. Public policies that promote a healthy lifestyle are essential. Some examples include creating smoke‐free zones, making dental treatment accessible, facilitating access to healthy food and allocating sufficient areas for physical exercise. Public health initiatives at retirement communities and community centres may also inspire seniors to choose better lifestyles. Future studies should focus on the underlying factors of these behaviours. The social, environmental and economic elements that lead to social inequalities in oral health must be addressed. To further improve our comprehension of social inequalities, it is important to consider the life‐course perspective and comprehend the cumulative effects of unfavourable socioeconomic status on oral health at various stages of life.

## 5 Conclusion

This study demonstrated that health‐related behaviours cluster among certain groups within the population. Clusters of health‐related behaviours was associated with having 20 teeth or more (functional dentition) among older English adults. These findings highlight the clustering pattern of behaviours. Implementing public health strategies that address these clusters, and the underlying social, economic, and environmental causes of oral health diseases is important.

## Author Contributions

Fatimah Alobaidi and Wael Sabbah contributed to the conceptualising of the study, designing the methodology, analysing and interpreting the data, editing and drafting the article. Wael Sabbah and Ellie Heidari participated in revising the manuscript and approving the final manuscript.

## Conflicts of Interest

The authors declare no conflicts of interest.

## Supporting information


**Table S1.** Sensitivity analysis for each behaviour with functional dentition.
