# Supplementary material for: Health‐Related Behaviour Clusters and Functional Dentition in Older People
Source: Gerodontology. 2025 Jan 8;42(3):396–404. doi: 10.1111/ger.12807 (PMC12344633; doi:10.1111/ger.12807)
Supplement: Supplementary file 1 — Table S1. Sensitivity analysis for each behaviour with functional dentition. [file GER-42-396-s001.docx]

**Health-related Behaviour Clusters and Functional dentition in Older People**

Fatimah Alobaidi^1^, Ellie Heidari^1^, Wael Sabbah^1^

^1^ Faculty of Dentistry, Oral & Craniofacial Sciences, King’s College London, London, United Kingdom

**Supplementary file: Statistical analyses**

Table 1: Sensitivity analysis for each behaviour with functional dentition.

| Behaviours | | OR | 95% CI |
| --- | --- | --- | --- |
| Physical activity | |  |  |
|  | No activity | 1.0 | - |
|  | Mild activity | 1.6 | (1.3, 2.1)*** |
|  | Moderate activity | 3.4 | (2.8, 4.1)*** |
|  | Vigorous activity | 6.6 | (5.3, 8.2)*** |
| Smoking | |  |  |
|  | Never | 1.0 | - |
|  | Former | 1.5 | (0.9, 2.4) |
|  | Current | 0.4 | (0.4, 0.5)*** |
| Alcohol | |  |  |
|  | Abstainer | 1.0 | - |
|  | Mild | 1.9 | (1.7, 2.2)*** |
|  | Moderate | 2.6 | (2.1, 3.3)*** |
|  | High | 2.9 | (1.1, 7.1)* |
| Fruits and vegetable | |  |  |
|  | < 5 portions | 1.0 | - |
|  | ≥ 5 portions | 1.2 | (1.0,1.4)* |

***p< 0.001.

*p< 0.05.
